# Hot Melt Extrusion for Improving the Physicochemical Properties of Polydatin Derived from *Polygoni cuspidati* Extract; A Solution Recommended for Buccal Applications

**DOI:** 10.3390/ph16091226

**Published:** 2023-08-30

**Authors:** Magdalena Paczkowska-Walendowska, Lidia Tajber, Andrzej Miklaszewski, Judyta Cielecka-Piontek

**Affiliations:** 1 Department of Pharmacognosy and Biomaterials, Poznan University of Medical Sciences, Rokietnicka 3, 60-806 Poznan, Poland; jpiontek@ump.edu.pl; 2 School of Pharmacy and Pharmaceutical Sciences, Trinity College Dublin, University of Dublin, D02 PN40 Dublin, Ireland; ltajber@tcd.ie; 3 Institute of Materials Science and Engineering, Faculty of Materials Engineering and Technical Physics, Poznan University of Technology, Jana Pawła II 24, 61-138 Poznan, Poland; andrzej.miklaszewski@put.poznan.pl

**Keywords:** hot melt extrusion, *Polygoni rhizoma et radix*, Kollidon^®^ VA64, Soluplus^®^, Kollicoat IR^®^, controlled release, dissolution, polydatin

## Abstract

Three different types of solid dispersions based on polyvinyl polymers and related copolymers (Kollidon^®^ VA64, Soluplus^®^ and Kollicoat IR^®^) comprising polydatin-rich *Polygoni cuspidati* extract were prepared by hot melt extrusion. The systems were characterized using X-ray powder diffraction, infrared spectroscopy as well as by polydatin release and in vitro permeability. Mucoadhesive tablets were prepared from the extrudates based on Kollidon^®^ VA64 and Soluplus^®^ to obtain a suitable pharmaceutical form, where (hydroxypropyl)methyl cellulose was added as a mucoadhesive agent. The tablets were evaluated in terms of the kinetics of polydatin release as well as their mucoadhesive properties. The best tabletability properties, polydatin release profile and adequate mucoadhesive properties were obtained by the formulation containing the Kollidon^®^ VA64-based extrudate, which makes it an excellent prototype for enhancing the release of poorly water-soluble compounds.

## 1. Introduction

*Polygoni cuspidati rhizoma et radix* is one of the most resveratrol- and polydatin-rich plant materials [1]. A variety of disorders is treated with *Polygonum cuspidatum*, also known as Hu Zhang in China, because of its antiviral, antibacterial, anti-inflammatory, neuroprotective, and cardioprotective properties. Importantly, the traditional use of *P. cuspidatum* as an oral hygiene agent is justified by its influence on bacterial viability and the virulence factors of *Streptococcus mutans* [2,3]. Such activity indicates the possibility of using extracts from this plant in dentistry. However, of relevance to topical use, the solubility of the active compounds and the achievement of a therapeutic concentration at the site of application are extremely important. Despite many advantages, the key active compounds in *P. cuspidatum* (polydatin and resveratrol) have low solubility [4], a barrier to ensuring the effectiveness of topical therapy.

Enhancement of solubility of poorly water-soluble active compounds has been one of the greatest challenges for researchers in the pharmaceutical industry. Amorphization has been shown as a successful and industry-relevant approach due to the increased apparent solubility of the compounds. A technique that can be effective in amorphizing drugs is hot melt extrusion (HME). HME is a solvent-free process requiring fewer unit steps than other disorder-inducing techniques. The formation of amorphous dispersions due to extrusion with selected polymers leads to crystallization inhibition, significantly improving the systems’ physical stability [5]. In addition, the dissolution rate of the active compounds from amorphous solid dispersions is often increased. This is the effect of limiting the energy needed to overcome the energy of the crystal lattice during the dissolution process. In addition, the presence of a hydrophilic carrier may improve the surface’s wettability to assist dissolution [6]. It should be remembered that the use of a high temperature may cause the decomposition of active ingredients, so it is also important to monitor the content of both active ingredients and decomposition products [7].

Amorphization of active compounds has also been used for substances of natural origin, including resveratrol. The resveratrol–Eudragit^®^ EPO system prepared by HME exhibited good miscibility and significant dissolution enhancement [8]. Eudragit EPO has also been used to make a solid dispersion with *Polygoni cuspidati* extract to improve the dissolution rate of resveratrol and emodin [9], while a hydrophobic–hydrophilic polymeric mix (based on a Eudragit RS and PEG 6000 combination) was employed to control the release of resveratrol. With the dispersive mixing and high shear forces of HME, resveratrol’s thermodynamic properties and dispersion were changed to improve its solubility and, thus, bioavailability to 140%, compared to resveratrol alone [10]. Moreover, HME was used to increase the chemical stability of resveratrol through the formation of eutectic systems with small molecules [11]. However, there are no further literature reports on the use of HME to simultaneously improve the properties of polydatin and resveratrol and obtain amorphous dispersions containing plant extracts rich in these two compounds. Bearing in mind the biological effects of both compounds and their synergism, it is justified to obtain amorphous dispersions comprising these two important polyphenols [12,13].

The success of HME is the application of appropriate carriers. Some of the most commonly used are polyvinyl-based copolymers. Due to the presence of various groups in the polymer’s backbone, polyvinyl polymers and related copolymers have a strong solubilizing capacity, which is their significant advantage. Polyvinyl alcohol–polyethylene glycol copolymer (Kollicoat IR^®^) is utilized for the production of immediate-release formulations, while polyvinyl pyrrolidone polymers (Kollidon^®^) and polymers containing polyvinyl chains are generally known to act as crystallization inhibitors, facilitating the formation and long-term stabilization of solid solutions (e.g., Soluplus^®^) or amorphous solid dispersions [14].

The work presented herein aims at investigating the impact of various polyvinyl-based copolymers on the molecular characteristics and pharmaceutical effects, such as the dissolution rate, of polydatin-rich *P. cuspidati* extract in amorphous solid dispersions. In addition, the mechanical properties and tableting behavior of the dispersions were tested. Finally, the usefulness of the developed buccal formulations was assessed, including polydatin release, ensuring product effectiveness and mucoadhesive properties keeping the product in the application place.

## 2. Results and Discussion

This study aimed to conduct a feasibility study on an extract of *Polygoni cuspidati* rhizome and root with known properties utilizing hot melt extrusion with subsequent formulation tests. According to the procedure described previously, lyophilized extract of *Polygoni cuspidati* rhizome and root was obtained [15]. The extract’s phytochemical and biological characteristics, such as its antioxidant and anti-inflammatory properties, were previously discussed [16].

**Figure 1:**
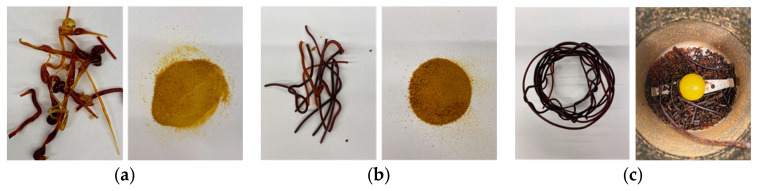
Macroscopic pictures of hot melt extrudates (as obtained and following grinding) based on (**a**) Kollidon^®^ VA64, (**b**) Soluplus^®^, (**c**) Kollicoat IR^®^.

The preparation of three types of solid dispersions (with Kollidon^®^ VA64, Soluplus^®^ and Kollicoat IR^®^) using the HME technique was first attempted. Importantly, in all cases the torque measured during extrusion was in the range 0.50–1.12 Nm, thus indicating no difficulty during processing. Figure 1 shows the morphology of all extrudates. It can be seen that the extrudates appear to be homogeneous and the outer surfaces are relatively smooth. However, the Kollicoat IR^®^-based extrudate was very pliable and could not be milled in either an agate mortar and pestle or a knife mill; therefore, it was decided not to proceed with this extrudate. Considering that the process temperature and screw speed significantly impact the post-processing drug content of the final extrudates [17], these parameters were considered in the context of thermal decomposition. The process temperature, which was 130 °C in the case of Kollidon^®^ VA64- and Soluplus^®^-based extrudates, was below the decomposition temperature of the active ingredients: resveratrol (melting point 261 to 263 °C) and polydatin (melting point 223–226 °C). This was confirmed by HPLC. Resveratrol and polydatin were retained at the same concentrations as in the original lyophilized extract: 143.09 µg and 952.59 µg, respectively, per 100 mg of extract. It was therefore deemed redundant to check the biological activity as this is linked to the amount of the intact, undegraded active in the extrudates.

**Figure 2:**
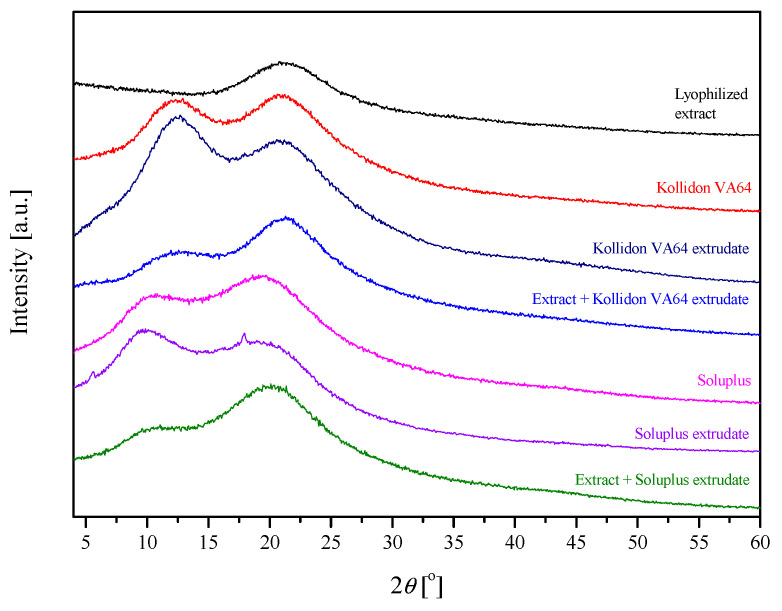
Diffractograms of extrudates.

The extrudates were then characterized by XRPD and FTIR-ATR. The X-ray diffractograms (Figure 2) of lyophilized extract show broad “halos”, which indicate their amorphous structure. Additionally, it was noted that the diffractograms of extruded samples show “halos” of the polymer matrix and the extract and their positions are presented in Table 1.

**Figure 3:**
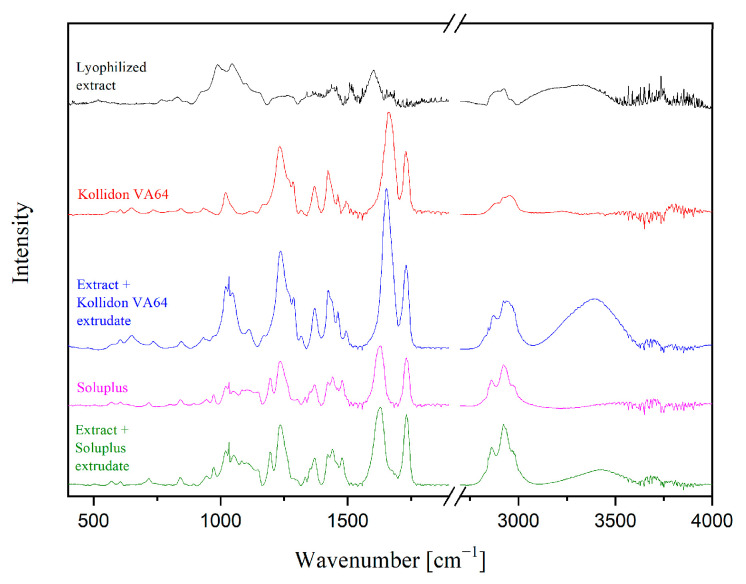
FTIR-ATR spectra of extrudates.

The *P. cuspidati* lyophilized extract has a broad band at 3300 cm^−1^, characteristic of stretching vibrations of H-bonded intermolecular OH groups, where the band at about 2950 cm^−1^ can be attributed to the asymmetric stretching vibration of a methyl group (Figure 3). The C=C stretching vibrations of the aromatic ring correspond to the peaks at 1450 cm^−1^, 1520 cm^−1^, and 1610 cm^−1^. Attributing the bands at 1520 cm^−1^ and 1450 cm^−1^ to the ketone C=O stretching and/or O-H bending vibrations is also possible. There are bands that are typical of the C-H vibrations in the aromatic rings between 950 and 1225 cm^−1^. Finally, the C-O-C stretching vibrations from ethers may be attributed to the band at approximately 1060 cm^−1^ [18]. Kollidon^®^ VA64 contains two hydrogen-bond receptor C=O groups, of the pyrrolidone ring (at 1668 cm^−1^) and vinyl acetate (at 1737 cm^−1^) [19]. The strong absorption bands in Soluplus^®^ reflect the carbonyl stretching vibrations of the caprolactam ring. The C=O frequencies of Soluplus^®^ at 1625 cm^−1^ and 1725 cm^−1^ are due to the presence of two different carbonyl groups, one peak at 1725 cm^−1^ for OC(O)CH_3_ or the ester group and the other at 1625 cm^−1^ for C(O)N or the amide group [20]. The spectral analysis suggests that the peaks present in the Kollidon^®^ VA64 or Soluplus^®^ are also present in the corresponding extrudates without significant shifts, and both extrudates also have the broad peak at 3300 cm^−1^ of the lyophilizate, which is indicative of miscibility of the components in the solid dispersions.

**Figure 4:**
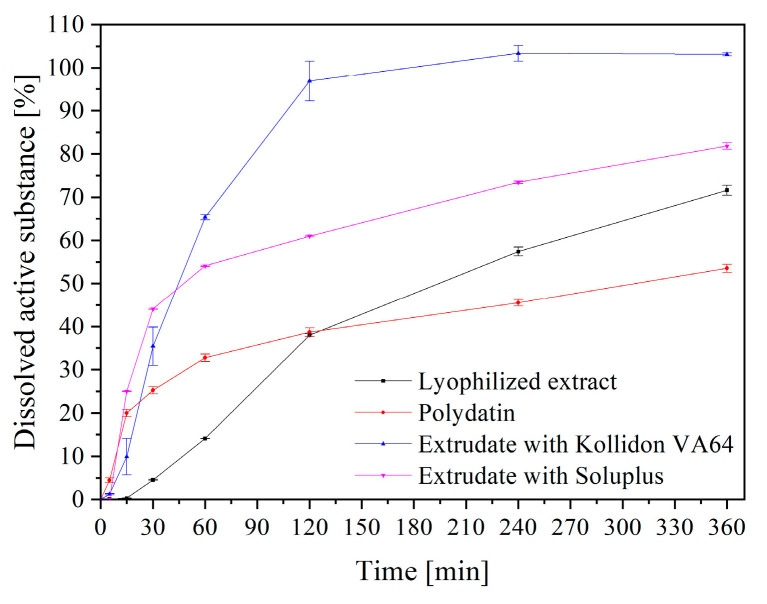
Dissolution profiles of polydatin from lyophilized extract and extrudates.

Another crucial factor that indicates how a formulation may act in terms of release in vivo is the in vitro dissolution testing of active substances from holt melt extrudates. The polydatin release profile from the ground hot melt extrudates based on Kollidon^®^ VA64 and Soluplus^®^ is shown in Figure 4. The dissolution profile of pure polydatin and the release profiles of polydatin from the lyophilized extract are also displayed for comparison. Due to its poor wettability and aggregation, only 50% of pure polydatin was dissolved within 6 h. The amount of polydatin released from the lyophilized extract reached almost 70% within 6 h, which may be due to the crystalline to amorphous transformation of this substance; however, the initial dissolution rate was slowed. The extrudate samples display a significantly improved dissolution curve, reaching about 50% dissolved in the first 30 min. Compared with the poor release of polydatin from the extract, the enhanced polydatin dissolution from the hot melt solid dispersions is most likely attributed to molecular dispersion of active compound in the polymers. Comparing the polydatin release profiles from both extrudates, we see that they also differ significantly. In the case of a Kollidon^®^ VA64-based extrudate, polydatin release is complete within 90 min. Kollidon^®^ VA64 is widely used in pharmaceutical technology, especially in hot melt extrusion, as an amorphous form stabilizer, which enhances the release of the active substances [6]. Therefore, better surface wetting, transformation of solid active substance from crystalline to amorphous form, and their dispersion into glassy matrix of amorphous polymer (Kollidon^®^ VA64) are the mechanisms underlying the increase in the dissolution rate [21]. Because the dissolution rate of the matrices prepared using Soluplus^®^ can be slowed by a viscous hydrogel layer that is formed on the surface of its particles, the release rate from hot-melt extrudates may be significantly reduced [22]. This phenomenon is clearly observed here, where with the same content of the extract in the products, the release of polydatin from the Soluplus-based system was slower after 1 h and reached only 80% release after 6 h.

In addition, permeability coefficients for active compounds using PAMPA-GIT model were established. The permeability coefficient for pure polydatin was 1.09 ± 0.07 × 10^−6^ cm/s, which confirms high permeability, classifying this compound as a BCS II active [23]. The amorphization of polydatin resulted in an improvement in solubility, which also boosted the compound’s permeability from the extrudates. The permeability coefficients for extrudates were 2.36 ± 0.02 × 10^−6^ cm/s and 1.78 ± 0.02 × 10^−6^ cm/s, respectively, for extrudates with Kollidon^®^ VA64 and Soluplus^®^.

In the next step, tablets containing Kollidon^®^ VA64- and Soluplus^®^-based extrudates were successfully prepared. A total of four formulations was obtained; two comprised the extrudates (formulations F1 and F3) and two contained the same amounts of ingredients but in the form of powders, which were controls and comparative formulations (formulations F2 and F4). In all formulations, HPMC was added as an excipient with mucoadhesive properties.

**Figure 5:**
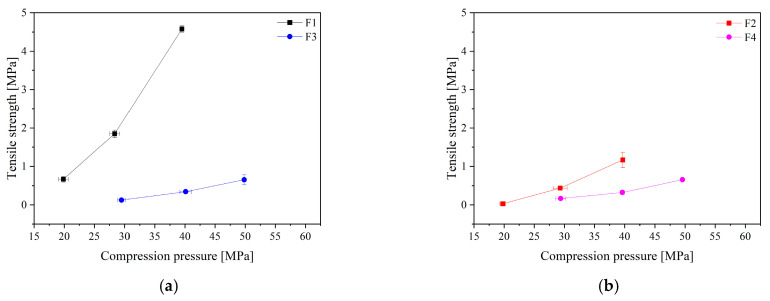

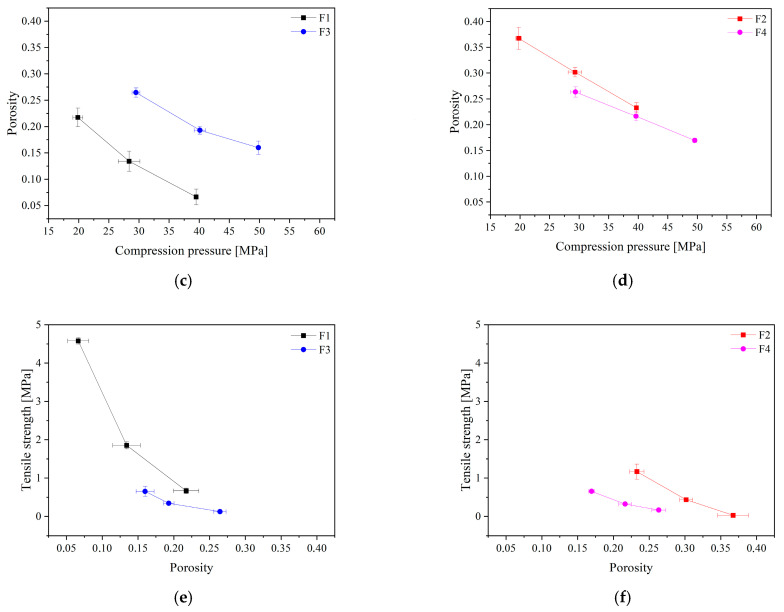
Tabletability (**a**,**b**), compressibility (**c**,**d**) and compactibility (**e**,**f**) of extrudates and powder systems, where F1—Kollidon/extrudate-, F2—Kollidon/powder-, F3—Soluplus/extrudate- and F4—Soluplus/powder-based tablets.

Firstly, tablets containing formulations F1–F4 were initially characterized in terms of tabletability, compressibility and compactibility (Figure 5). The tabletability of the tablets decreased in the following order: F1 > F2 > F3 > F4 (Figure 5a,b). In general, higher tensile strength tablets were obtained when extrudates were tableted compared to powder tablets. Additionally, tablets based on Kollidon^®^ VA64 have better tabletability properties than those based on Soluplus^®^. Tablets with Soluplus^®^-based-extrudates have slightly lower tensile strengths compared to the tablets made using physical mixtures. This could be due to small changes in the bonding area (reflected by the compressibility) and/or changes in the bonding strength per unit bonding area (reflected by the compactibility) [24]. When we analyzed the compressibility profiles (Figure 5c,d), we obtained slightly lower porosities for the tablets with Kollidon^®^ VA64 extrudate, indicating higher interparticulate bonding areas, which could explain the higher tabletability for this formulation. No significant differences in compressibility were detected for formulations with Soluplus^®^ in the extrudate and powder forms. The Ryshkewitch equation states that tablet tensile strength decreases exponentially with increasing porosity, and compactibility describes this relationship. By analyzing compactibility, the origin of the differences in tablet tensile strength for the Kollidon^®^ VA64-based formulation was detected, since powders showed lower interparticulate bonding strength at a specific porosity compared to the extrudate. The order of decreasing compatibility appears to be as follows: F1 > F2 > F3~F4 (Figure 5c,d). It can also be seen from Figure 5c,d that the tablet tensile strength decreases exponentially with increasing porosity, which fits the Ryshkewitch equation where T_S0_ is the extrapolated tensile strength at zero porosity. This parameter, T_S0_, is often used to compare bond strength. The T_S0_ of F2, F4 was 3.3 and 1.6 MPa, respectively; thus, the bonding strength of powders was poor. The T_S0_ of extrudates were higher, 6.2 and 1.8, respectively, for F1 and F3. The enhanced bond strength thus shows that the HME technique can enhance this metric. This may be because components came into closer contact and with more consistency during melting and extrusion. It can also be the result of the transformation active compounds from the crystalline state to the amorphous dispersion [25]. Based on the above parameters, the best tablet properties were obtained for formulation F1 (Kollidon^®^ VA64-based extrudate).

**Figure 6:**
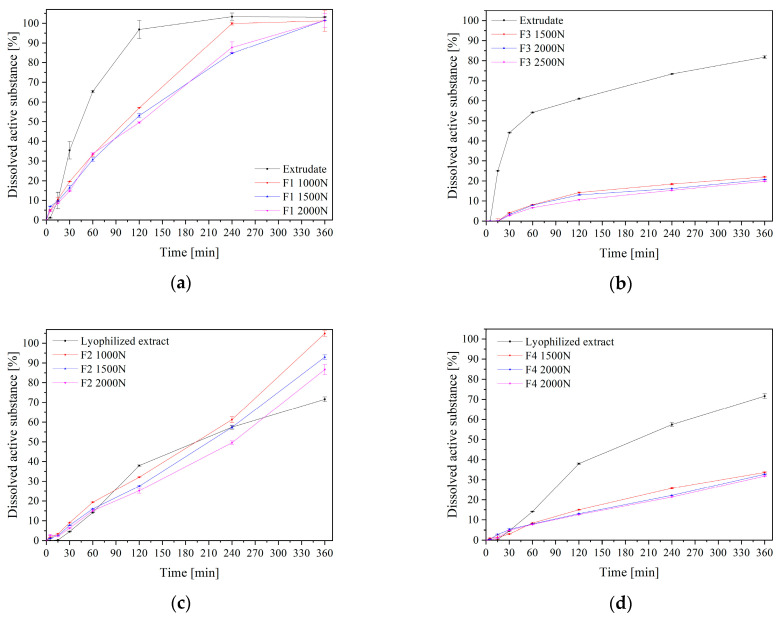
Dissolution profiles of extrudate-based tablets (**a**,**b**) and powder-based tablets (**c**,**d**) where F1—Kollidon/extrudate-, F2—Kollidon/powder-, F3—Soluplus/extrudate- and F4—Soluplus/powder-based tablets.

**Figure 7:**
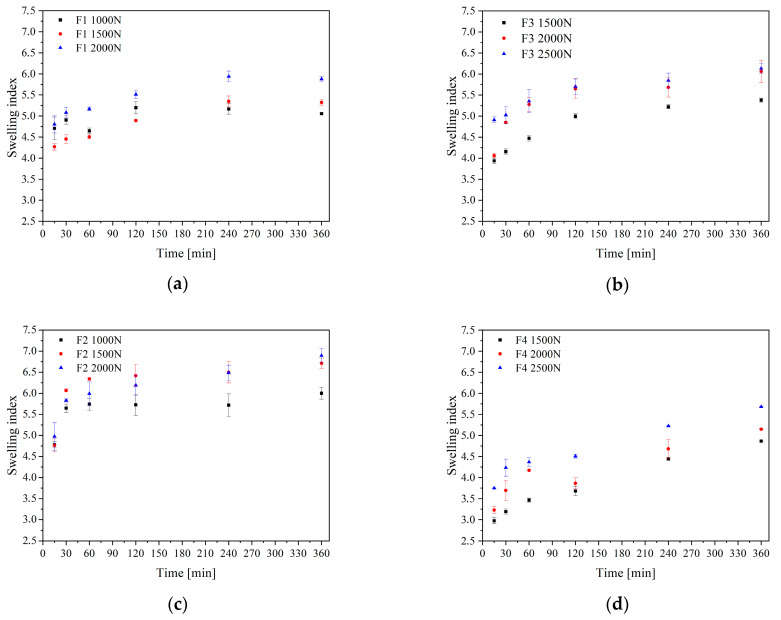
Swelling index of extrudate-based tablets (**a**,**b**) and powder-based tablets (**c**,**d**), where F1—Kollidon/extrudate-, F2—Kollidon/powder-, F3—Soluplus/extrudate- and F4—Soluplus/powder-based tablets.

In the next step, the dissolution rates of polydatin from the F1–F4 formulation were assessed (Figure 6). As previously mentioned, a higher rate of polydatin dissolution from both the lyophilized extract and the extrudates in the particulate forms was seen, which is associated with the transition from the crystalline to the amorphous form (Figure 4). Extrudates based on Kollidon^®^ VA64 and Soluplus^®^ that were made at different compression pressures were examined for the polydatin dissolution profiles. Nevertheless, the dissolution rates from the tablets made at those different compression pressures were comparable (in all cases f_1_ was below 10 and f_2_ was above 50). However, disparities were seen in the swelling index, which increased with the increase in the compression force (Figure 7). Importantly, polydatin release from the extrudate formulations based on Kollidon^®^ VA64 was quick, even quicker than release from tablets based on physical mixtures, where a slow and controlled release was observed (Figure 6c). The delayed release from the tablets with the Soluplus^®^-based extrudate was further augmented by the addition of HPMC, an additional agent causing delayed release by forming a highly viscous coating on the tablet surface (Figure 6b,d). The release from Soluplus^®^-based tablets reached a maximum of 30% after 6 h. Despite achieving a controlled release, the likelihood of the mucoadhesive tablet remaining on the oral mucosa for this long are low, due to reasons of patient comfort as well as a slow disintegration of the tablet itself.

The kinetics of polydatin release shows differences when comparing tablets based on extrudate and those based on physical mixtures. Table S1 (Supplementary Material) contains mathematical models that describe the kinetics of polydatin release from formulations F1–F4. According to the information provided above, the release of polydatin from powder-based tablets (formulations F2 and F4) is significantly delayed and controlled, and the release of polydatin follows zero-order kinetics, which indicates that the polydatin release is consistent over time. The entire release of the bioactives should take place within 2 h if mucosal application within the oral cavity is desired, because a longer stay of the tablet attached to the mucous membrane may be irritating for the patient. The polydatin release from tablets comprising the Kollidon^®^ VA64 extrudate is the most favored in this regard. Formulation F1 displayed behavior following the Korsmeyer–Peppas model with ”n” values in the range of 0.45 to 0.89 indicating that the release approximates the non-Fickian diffusion release mechanism [26]. According to the literature data, the relative complexity of the created formulations may suggest that there are multiple mechanisms controlling the active compounds release. These mechanisms include polymer erosion, swelling, and dissolution, all of which were employed in the release process [27]. Similar dependencies have been described previously [28]. In contrast, the release from Soluplus^®^ extrudate-based tablets was greatly delayed in time, reaching only 20% polydatin release after 6 h. It has been previously described that a higher concentration of Soluplus^®^ cause the medium’s viscosity to increase and a slower rate of active substance dissolution [29]. Traditional Chinese medicine (TCM) has been widely used in China for thousands of years to treat and prevent diseases. TCM has been proven safe and effective, is considered as one of the important types of complementary and alternative medicine, and receives increasing attention worldwide. The dried root of Polygonum cuspidatum Sieb. et Zucc. (also known as “Hu Zhang” in Chinese) is one of the medicinal herbs listed in the Pharmacopoeia of the People’s Republic of China. Hu Zhang is widely distributed across the world. It can be found in Asia and North America and is used as folk medicine in countries such as Japan and Korea. In China, Hu Zhang is usually used in combination with other TCM herbs. The therapeutic uses of those Hu Zhang-containing TCM prescriptions or formulations are for treating cough, hepatitis, jaundice, amenorrhea, leucorrhea, arthralgia, burns and snake bites. Recent pharmacological and clinical studies have indicated that Hu Zhang has antiviral, antimicrobial, anti-inflammatory, neuroprotective, and cardioprotective functions.

**Figure 8:**
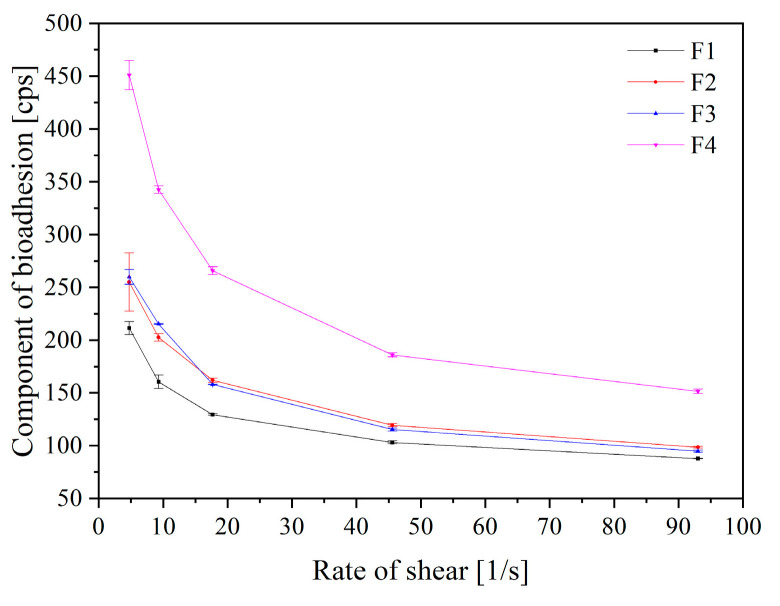
Component of bioadhesion of extrudate-based and powder-based tablets, where F1—Kollidon/extrudate-, F2—Kollidon/powder-, F3—Soluplus/extrudate- and F4—Soluplus/powder-based tablets.

Finally, rheological tests were used to assess the mucoadhesive qualities of formulations F1–F4 (Figure 8). HPMC was used as a mucoadhesive agent in all systems. Given that HPMC is a non-ionic polymer, the media pH had no bearing on how well it adhered to the mucosa. Therefore, HPMC serves as the primary mucoadhesive agent in the mixes mentioned. This polymer has several hydroxyl groups, which enable it to interact physically (including through hydrogen bonds) with the elements of mucus [30,31]. Mucoadhesive properties of the tablets decreased as follows: F4 > F2 > F3 > F1, which indicates that the mucoadhesive properties of Soluplus^®^ are greater than those of Kollidon^®^ VA64. The literature indicates that higher concentrations of Soluplus^®^ may remain of interest for the production of mucoadhesive gelling systems. In fact, the micellar suspension of Soluplus^®^ can change into a weak gel on the ocular surface at high concentrations, which may extend the formulation’s permanence period while providing gentle resistance to blinking and finally having a lubricating effect [32]. It is therefore worth considering using Soluplus^®^ to apply a single polymeric material to achieve both nano-micelle and gelling properties. The mucoadhesive properties of Kollidon^®^ VA64 are slightly weaker; however, this polymer is described in the literature as a film-forming agent [33]. This also confirms the observations from the dissolution study, because forming a thin layer does not allow rapid diffusion of the drug to the fluid and it perhaps also acts as a binder, protecting the formulation against fast disintegration.

Additionally, the residence time of the tablets was examined to further understand their mucoadhesive activity upon contact with the saliva-simulating media. All formulations subjected to the test immediately adhered to the tissue, grew gradually when they made contact with the acceptor medium, and displayed no signs of disintegration at any time throughout the test. The contact time of tablets F2 and F4 with the mucosal surface was preserved over the period of the 240-min test, but formulae F1 and F3 separated from the tissue after 210 and 220 min, respectively (Table 2), supporting the findings of rheological tests.

## 3. Materials and Methods

### 3.1. Plant Material

Plant raw material, *Polygonum cuspidatum* rhizome and root, was purchased from Herbapol Cracow (Cracow, Poland) (Lot No. 010918).

### 3.2. Chemicals and Reagents

Resveratrol (≥99%, HPLC) and polydatin (≥95%, HPLC) were obtained from Sigma-Aldrich (Poznan, Poland). Excipients, such as Kollidon^®^ VA64, Soluplus^®^, and Kollicoat IR^®^, were supplied by BASF (Warsaw, Poland), and (hydroxypropyl)methyl cellulose (HPMC) with average Mn ~ 90.000 (~15.000 cP) and magnesium stearate were supplied by Sigma-Aldrich (Poznan, Poland). Mucin from a porcine stomach was obtained from Sigma-Aldrich (Poznan, Poland). HPLC grade acetonitrile and water were obtained from Merck. High-quality pure water and ultra-high-quality pure water were prepared using a Direct-Q 3 UV Merck Millipore purification system (Darmstadt, Germany).

### 3.3. Preparation of Solid Dispersions

#### 3.3.1. Preparation of Lyophilized Extract

A quantity of 5.0 g of dried rhizome and root of *Polygonum cuspidatum* was extracted four times with a 50.0 mL ethanol–water mixture (7:3 *v*/*v*) for 20 min at 70 °C on an ultrasound-assisted water bath, Elmasonic S180H (Elma, Singen, Germany). The obtained extracts were collected and concentrated on a vacuum evaporator at a temperature of 50 °C to a volume of 50.0 mL (BÜCHI Rotavapor R-210) obtaining at that time a drug−extract ratio (DER) of 1:10 [16]. The extract was then frozen and lyophilized (CHRIST 1–4 LSC, Osterode am Harz, Germany). The temperature inside the product was estimated to be −4 °C and the condensation temperature was set to −48 °C. The freeze-drying was conducted at reduced pressure (1.030 mbar) for 48 h.

#### 3.3.2. Hot Melt Extrusion (HME)

Extrusion was performed on a HAAKE MiniCTW micro-conical twin screw extruder (Thermo Scientific, Karlsruhe, Germany). The lyophilized extract (15% by weight) and three types of polyvinyl-based copolymers (Table 3) were mixed with a pestle and mortar and subsequently fed manually into the hopper of the extruder at barrel temperature and the screw speed given in Table 1. The extrudates were collected, softly ground manually with a pestle and mortar, passed through an 80-mesh sieve, and kept in a desiccator at room temperature for further analysis.

#### 3.3.3. Powder X-ray Diffraction (PXRD)

The crystallographic structure of the extrudates was analyzed by an X-ray diffraction (XRD, Panalytical Empyrean, Almelo, The Netherlands) with the copper anode (CuKα—1.54 Å) at a Brag–Brentano reflection mode configuration with 45 kV and 40 mA parameters. The measurement parameters were set up for 3–60° with a 45 s per step 0.05° in all cases.

#### 3.3.4. Fourier Transform Infrared Spectroscopy with Attenuated Total Reflectance (FTIR-ATR)

The FTIR-ATR spectra were measured between 400 cm^−1^ and 4000 cm^−1^, with a resolution set to 1 cm^−1^, with a Shimadzu IRTracer-100 spectrometer equipped with a QATR-10 single bounce—diamond extended range and LabSolution IR software (version 1.86 SP2, Shimadzu, Kyoto, Japan).

#### 3.3.5. Determination of Active Components Content

The contents of the main active compounds (polydatin, resveratrol, emodin, and parietin; Figure S1, Supplementary Material) were determined by using the modified HPLC-Diode-Array, described previously by Paczkowska-Walendowska et al. [15,16]. Briefly, separations were performed on a LiChrospher RP-18 column with a 5 μm particle size, 250 × 4 mm (Merck, Darmstadt, Germany). The mobile phase was composed of formic acid 0.1% (A) and acetonitrile (B) with a gradient elution of 0–20 min, 15–20% B; 20–40 min, 20–40% B; 40–60 min, 40–100% B; 60–65 min, 100% B; 65–70 min, 15% B. The detection was performed at a wavelength (*λ*_max_) of 290 nm. The flow rate of the mobile phase was set at 1.0 mL/min, and the column temperature at 30 °C.

#### 3.3.6. Dissolution/Release Studies

Release studies of polydatin from extrudates were performed using an Agilent 708-DS dissolution apparatus. A conventional paddle method with 50 rpm stirring was used at 37 ± 0.5 °C. Extrudates were dissolved in 300 mL of an artificial saliva solution made of potassium chloride (1.20 g), sodium chloride (0.85 g), di-potassium hydrogen orthophosphate (0.35 g), magnesium chloride (0.05 g), calcium chloride (0.20 g), xylitol (20.0 g) and water up to 1 L. The pH of the solution was then adjusted to 6.8 using 1 M HCl. Liquid samples were taken at specific intervals, and an equal volume of temperature-stabilized medium was added in their place. The samples were filtered using a nylon membrane filter with a mesh size of 0.45 microns. The amounts of polydatin in the filtered acceptor solutions were determined using the HPLC method described in Section 3.3.5. Sink conditions were preserved in the studies.

The model proposed by Moore and Flanner, which is based on two-factor values, f_1_ and f_2_, was used to compare the dissolution profiles [34].

#### 3.3.7. Permeability Studies

The PAMPA^TM^ (parallel artificial membrane permeability assay) gastrointestinal tract (GIT) assay (Pion Inc., Billerica, MA, USA) was used to examine the permeability of the active compound (polydatin), released from the extrudates, via artificial biological membranes. Donor solutions (artificial saliva solution at pH 6.8) were used to dissolve the extrudates. Acceptor Prisma buffer with a pH of 7.4 was added to the acceptor plates. The plates were assembled and incubated for 60 min at 37 °C with 50 rpm of continuous stirring. Each experiment was run again at least three times. The above-mentioned HPLC procedure was used to calculate the quantity of penetrated polydatin. The apparent permeability coefficients (*P*_app_) were calculated from the following equation:

$$P_{app}=\frac{-ln\left(1-\frac{C_{A}}{C_{equilibrium}}\right)}{S\times \left(\frac{1}{V_{D}}+\frac{1}{V_{A}}\right)\times t}$$

where *V_D_* is the donor volume, *V_A_* is the acceptor volume, *C_equilibrium_* is the equilibrium concentration $C_{equilibrium}=\frac{C_{D}\times V_{D}+C_{A}\times V_{A}}{V_{D}+V_{A}}$, *C_D_* is the donor concentration, *C_A_* is the acceptor concentration, *S* is the membrane area, and *t* is the incubation time (in seconds).

### 3.4. Tableting

A laboratory-scale, single punch NP-RD10A tablet press was used to compress tablets that were flat-faced and 8 mm in diameter (Natoli, Saint Charles, MO, USA). Utilizing a variety of compaction forces between 1000 and 3000 N (corresponding to compression pressure in the range of 20 to 60 MPa), we evaluated the compaction characteristics of the tablets. When the desired compaction force was reached, the pressure was released. Table 4 lists the ingredients of the formulations.

#### 3.4.1. Tablet Characterization

Following the tablet preparation, the tablets were weighed. The uniformity of the tablet mass was regulated using a method described in Ph.Eur. 9th. Additionally, the diameter and thickness of 20 randomly selected tablets were measured using a manual vernier caliper. Following all measurements, mean values and standard deviations (SD) were calculated.

The tablet hardness was determined using the procedures outlined in Ph.Eur. 9th, and the PTB-M manual tablet hardness testing device (Natoli, Saint Charles, MO, USA) was employed. Tablet hardness reported in this work is a mean value with a standard deviation of six measurements.

Tensile strength (*σ*) values were calculated on the basis of the breaking force (*F*) values (N), where *d* is the diameter of the tablet (mm) and *h* is the thickness of the tablets (mm) [35].

$$\sigma =\frac{2F}{\pi dh}$$

The solid fraction (SF) was calculated by the equation, where *W_t_* is the weight of the tablet (mg), *v* is the tablet volume, and *ρ_true_* is the powder’s true density (g/cm^3^).

$$F=\frac{W_{t}}{\rho_{true}v}$$

The tablet porosity (*ε*) was calculated from the SF using the following equation:

ε=1−SF

The compactibility of powders was also analysed with the Ryshkewitch equation:

$$\varepsilon =\varepsilon_{0}\times exp(-b\times P)$$

$$T_{S}=T_{0}\times exp(-k\times \varepsilon )$$

where *P* is the porosity of the powder when *P* = 0; *b* is a constant which is inversely proportional to the yield strength of materials; *T_s_* and *T*_0_ are the tablet tensile strength and limiting tablet tensile strength at zero porosity, respectively, and *k* is an empirical constant [25].

#### 3.4.2. In Vitro Release Studies

In vitro release studies of polydatin from prepared tablets were performed according to the methodology described in Section 3.3.6. The resulting active compound release profiles were fitted to the following mathematical models to study the release kinetics: [36]: zero-order equation: F=k×t, first-order equation: nF=k×t, Higuchi equation: $F=kt^{1/2}$, Korsmeyer–Peppas equation: $F=kt^{n}$, where *F*—the fraction of release drug, *k*—the constant associated with the release, and *t*—the time.

#### 3.4.3. Swelling Index

Each tablet was individually weighed and placed in a 25 mL beaker that contained 10 mL of an artificial saliva solution at pH of 6.8 and at 37 ± 0.5 °C. The tablets were taken out, cleaned with filter paper, and reweighed at the pre-set time intervals (15, 30, 60, 120 and 240 min). The swelling index was calculated by using the following formula:

$$SI=\frac{W_{2}-W_{1}}{W_{1}}$$

where *SI* is the swelling index, *W*_1_ is the initial weight of the tablet, *W*_2_ is the weight of the tablet after the particular swelling time interval. Each experiment was performed in triplicate.

#### 3.4.4. In Vitro Assessment of Mucin-Biopolymer Bioadhesive Bond Strength

A viscometric method was used to quantify mucin-polymers’ (mucin-tableting mixtures’) bioadhesive bond strength. The evaluation was performed according to the method described previously [15].

#### 3.4.5. Determination of the Residence Time

On modified testing equipment, the residence duration of tablets to regenerated cellulose membrane simulating porcine buccal mucosa was assessed in accordance with earlier studies. [15]. In brief, the medium was a pH 6.8 artificial saliva solution kept at a constant 37 ± 0.5 °C. Each tablet was in contact with the cellulose foil for 5 s by applying finger pressure. Over a period of 2 h, the mixture had to be separated from the foil that simulated the mucosal tissue. Studies were carried out in triplicate.

### 3.5. Statistical Analysis

The statistical analysis was performed using Statistica 13.3 software. The results’ normal distribution was checked using the Shapiro–Wilk test. The variations between the mean values were examined using the ANOVA test together with the post hoc Tukey’s test for multiple comparisons. Differences across groups were considered significant at *p* < 0.05.

## 4. Conclusions

Hot melt extrusion allowed amorphous dispersions of polydatin to be obtained. These systems significantly improved release of the active. Extrudates comprising the *P. cuspidati* extract can be prepared at temperatures around 130 °C, which is below the decomposition temperature of the active compounds. Finally, amorphization of bioactives upon extrusion was associated with improved polydatin dissolution. The dissolution data revealed that Soluplus^®^ led to a dissolution improved by 20% compared to the lyophilized extract, while the addition of Kollidon^®^ VA64 resulted in the complete dissolution of polydatin.

The use of extrudates increases tabletability and compressibility properties and tablets with a porous structure, while maintaining their high tensile strength, were produced. Based on these results, a combination of Kollidon^®^ VA64 and HPMC in a tablet formulation significantly improved the dissolution properties, allowing for control of the process.

Thus, the best tabletability properties and an increased polydatin release, while maintaining sufficient mucoadhesive properties, were provided by the formulation containing the Kollidon^®^ VA64-based extrudate.

## Figures and Tables

**Table 1:** Position of XRPD “halos”.

Sample	Lyophilized Extract	Kollidon^®^ VA64	Extract + Kollidon^®^ VA64 Extrudate	Soluplus^®^	Extract + Soluplus^®^ Extrudate
(1) Peak position [2θ]	-	12.46	12.59	10.75	11.09
(2) Peak position [2θ]	21.37	21.02	21.32	19.43	20.12
Matrix peak position displacement [2θ]	-	-	(1) 0.12 (2) 0.30	-	(1) 0.34 (2) 1.47
Matrix peak position displacement [Å]	-	-	(1) −0.07 (2) −0.06	-	(1) −0.24 (2) −0.16

**Table 2:** Residence time of extrudate- and powder-based tablets.

Formulation	F1	F2	F3	F4
Residence time (min)	210 ± 10	>240	220 ± 10	>240

**Table 3:** Composition of extrudates and process parameters.

Chemical Name	Trade Name	Composition of Extrudates	Processing Temperature	Screw Speed	Extruder Torque
Polyvinylpyrrolidone- co-vinyl acetate	Kollidon^®^ VA64	Extract 15% Kollidon^®^ VA64 75% Glycerine 10% as a plasticizer	130 °C	150 rpm	0.50 Nm
Polyvinyl caprolactam–polyvinyl acetate–polyethylene glycol graft copolymer	Soluplus^®^	Extract 15% Soluplus^®^ 85%	130 °C	50 rpm	1.12 Nm
Polyvinyl alcohol–polyethylene glycol copolymer	Kollicoat IR^®^	Extract 15% Kollicoat IR^®^ 85%	160 °C	150 rpm	0.73 Nm

**Table 4:** Compositions of formulations.

	Formulation 1 (F1)	Formulation 2 (F2)	Formulation 3 (F3)	Formulation 4 (F4)
	Content (mg) per 1 tablet			
Lyophilized extract (15%)/Kollidon^®^ VA64-extrudate	100.0			
Lyophilized extract/Soluplus^®^-extrudate			100.0	
Lyophilized extract		15.0		15.0
Kollidon^®^ VA64		85.0		
Soluplus^®^				85.0
HPMC 15.000 cP (20%)	20.0	20.0	20.0	20.0
Stearate magnesium	1.0	1.0	1.0	1.0
Total	121.0	121.0	121.0	121.0

## Data Availability

Data are contained within the article.

## Supplementary Materials

The following supporting information can be downloaded at: https://www.mdpi.com/article/10.3390/ph16091226/s1, Table S1. Mathematical models of release profiles of formulations F1–F4. Figure S1. Chromatogram of *Polygoni cuspidati* extract.
