# Supplementary material for: Hot Melt Extrusion for Improving the Physicochemical Properties of Polydatin Derived from Polygoni cuspidati Extract; A Solution Recommended for Buccal Applications
Source: Pharmaceuticals (Basel). 2023 Aug 30;16(9):1226. doi: 10.3390/ph16091226 (PMC10535885; doi:10.3390/ph16091226)
Supplement: Supplementary file 1 [file pharmaceuticals-16-01226-s001.zip › pharmaceuticals-2568213-supplementary.pdf]

Hot Melt Extrusion for Improving the Physicochemical Properties of Polydatin Derived from *Polygoni cuspidati* Extract; A Solution Recommended for Buccal Applications

Supplementary material

Table S1. Mathematical models of release profiles of formulations F1-F4.

Formulation		Mathematical model							
No.	Compression pressure	Zero-order kinetic		First-order kinetic		Higuchi kinetic		Korsmeyer-Peppas kinetic	
		K	R^2	K	R^2	K	R^2	R^2	n
F1	1000N	**18.04**	**0.92**	0.67	0.60	18.86	0.87	**0.95**	**0.64**
	1500N	**17.13**	**0.96**	0.55	0.62	12.90	0.87	**0.95**	**0.62**
	2000N	**17.44**	**0.96**	0.58	0.63	13.06	0.86	**0.96**	**0.63**
F2	1000N	**16.85**	**0.99**	0.68	0.76	11.33	0.75	0.98	0.64
	1500N	**15.21**	**0.99**	0.69	0.77	10.17	0.74	0.97	0.64
	2000N	**13.86**	**0.99**	0.65	0.80	9.21	0.73	0.97	0.60
F3	1500N	**3.86**	**0.90**	0.52	0.69	2.99	0.88	**0.90**	**0.48**
	2000N	**3.56**	**0.91**	0.51	0.70	2.76	0.88	**0.90**	**0.46**
	2500N	**3.41**	**0.94**	0.51	0.75	2.55	0.86	**0.91**	**0.45**
F4	1500N	**5.77**	**0.98**	0.59	0.78	4.12	0.82	0.93	0.52
	2000N	**5.28**	**0.99**	0.57	0.68	3.79	0.83	0.88	0.52
	2500N	**5.22**	**0.99**	0.56	0.76	3.72	0.82	0.93	0.49

the most fitting mathematical model is shown in bold.

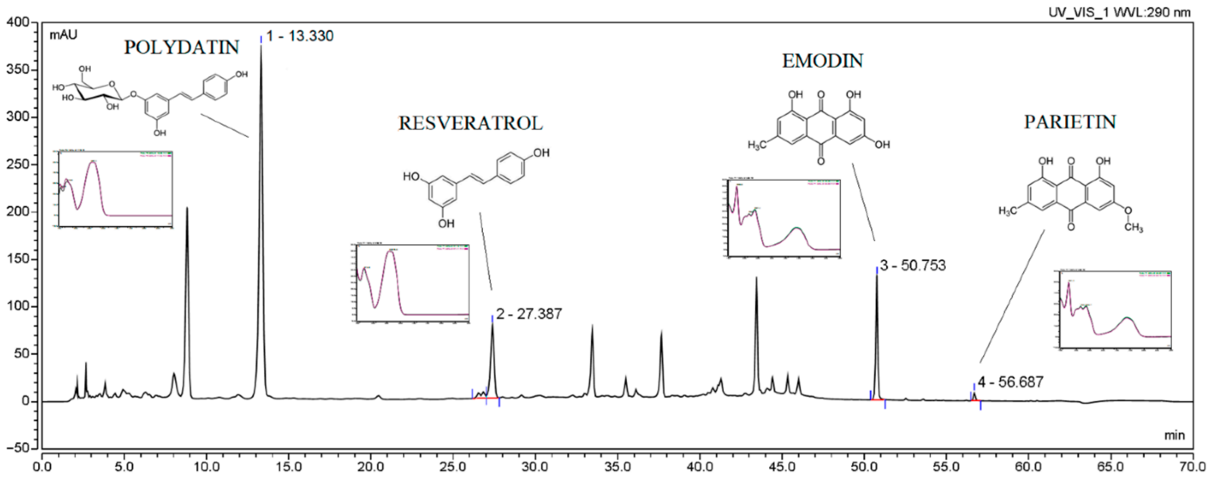

Figure S1. Chromatogram of *Polygoni cuspidati* extract.
